# Stress-Testing Meta-Analyses: A Reproducible Python Framework for Robustness Assessment of Continuous Outcomes

**DOI:** 10.7759/cureus.116020

**Published:** 2026-09-09

**Authors:** Sadia Nadeem

**Affiliations:** 1 General Practice, Punjab Medical College, Faisalabad, PAK

**Keywords:** continuous outcomes, hedges g, heterogeneity, meta-analysis, python, reproducibility, robustness, sensitivity analysis

## Abstract

Meta-analyses of continuous outcomes often summarize evidence as a single pooled effect estimate, but the stability of that estimate can depend on the effect-size calculation, heterogeneity estimator, confidence-interval method, and influence of individual studies. This technical report presents a reproducible Python framework for stress-testing pairwise meta-analyses of continuous outcomes from independent two-group comparisons. The workflow accepts study-level means, standard deviations, and sample sizes; validates the input; calculates Hedges g and its approximate sampling variance; fits fixed-effect and random-effects models; compares DerSimonian-Laird and Paule-Mandel estimates of between-study variance; calculates a prediction interval independently of the pooled-effect confidence interval (CI); applies a conservative Hartung-Knapp-Sidik-Jonkman (HKSJ)-style confidence-interval adjustment; performs leave-one-out influence analysis; identifies the study producing the largest absolute change in the pooled estimate; and performs an Egger-type regression for funnel-plot asymmetry when at least 10 studies are available. The demonstration uses a synthetic dataset of 12 independent studies. The primary random-effects estimate was Hedges g = -0.249 (95% CI, -0.455 to -0.043; tau-squared = 0.091), with a 95% prediction interval of -0.961 to 0.463. The HKSJ-style CI was wider (-0.494 to -0.003), while the Paule-Mandel model produced a similar pooled estimate (Hedges g = -0.248; 95% CI, -0.467 to -0.028). All leave-one-out estimates retained a negative direction, and Study 07 produced the largest absolute change (0.074 Hedges g). The Egger-type intercept test was not statistically significant (p = 0.352). The framework is intended to make statistical fragility explicit and reproducible while complementing, rather than replacing, established meta-analysis software.

## Introduction

Meta-analysis combines quantitative evidence from multiple studies to estimate an overall effect, but a pooled estimate should not be interpreted independently of the assumptions used to obtain it. In a random-effects model, the pooled estimate represents an average underlying effect, while between-study heterogeneity contributes to the uncertainty and to the range of effects that may be expected in other settings [1,2]. A fixed-effect model assumes that the studies estimate one common underlying effect, whereas a random-effects model allows the underlying effect to differ between studies. Therefore, the two models may produce different pooled estimates and measures of uncertainty.

Heterogeneity can be described using Cochran's Q, between-study variance (tau-squared), and I-squared (I²), but these measures address different aspects of variation. Q evaluates whether observed differences are greater than expected from sampling error, whereas I² describes the proportion of observed variability attributed to between-study heterogeneity under the specified model [3,4]. Neither measure alone establishes whether the pooled estimate is robust to alternative analytical choices or individual studies.

For continuous outcomes, the standardized mean difference (SMD) can combine studies that assess the same construct with different measurement scales. Hedges g applies a small-sample correction to the standardized mean difference [5]. Random-effects results can vary according to the estimator used for between-study variance. DerSimonian-Laird is widely used, whereas alternative estimators such as Paule-Mandel can produce different estimates of between-study variance and consequently different weights and uncertainty [6,7].

Small-sample inference is another source of analytical sensitivity. Hartung-Knapp-type procedures modify inference for the pooled effect using a t distribution and an adjustment to the estimated variance [8,9]. A confidence interval describes uncertainty around the pooled average effect, whereas a prediction interval describes the plausible range of a true effect in another comparable setting. Prediction intervals address a different inferential target by incorporating between-study heterogeneity to describe a plausible range for a true effect in another setting [2,10]. Influence diagnostics can identify studies that disproportionately affect a fitted meta-analytic model [11]. Tests related to funnel-plot asymmetry or small-study effects should be interpreted cautiously because asymmetry can have multiple causes, and statistical power may be limited when few studies are available [12].

The objective of this technical report is to provide a transparent, reproducible, and reusable Python workflow for stress-testing continuous-outcome meta-analyses. The main purpose of the computational layer is auditability: it makes data validation, effect-size calculation, model assumptions, sensitivity analyses, influence assessment, and reportable robustness conclusions explicit. Established R packages such as metafor and meta provide broad meta-analysis functionality; this workflow complements rather than replaces these tools by making data validation and stress tests transparent and reproducible [13,14].

## Technical report

Framework architecture

The workflow follows a prespecified sequence: data input and direction-of-benefit definition; input validation; effect-size and variance calculation; primary random-effects synthesis; planned stress tests; automated robustness assessment; and export of machine-readable results. Figure 1 summarizes the workflow. The demonstration uses synthetic data so that every calculation can be rerun without access to a clinical dataset.

**Figure 1:**
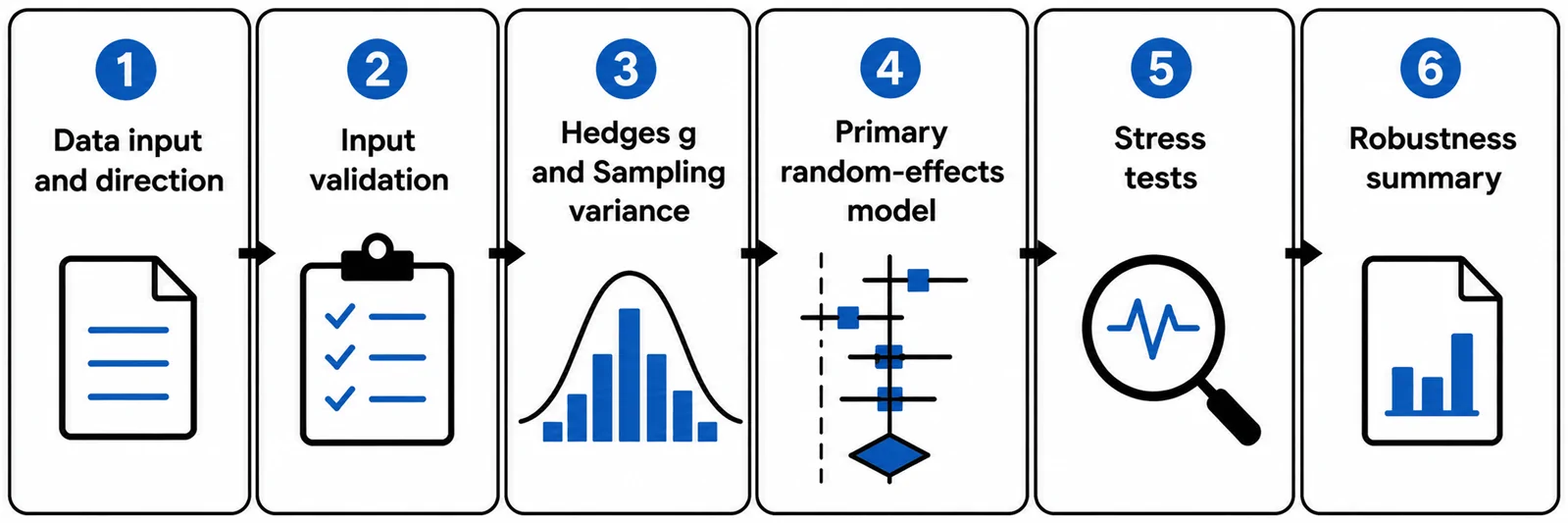
Stress-testing workflow for continuous-outcome meta-analysis The workflow separates data input, validation, effect-size calculation, primary synthesis, prespecified stress tests, and robustness reporting. Stress tests include fixed-effect model, Paule-Mandel tau-squared, HKSJ-style confidence interval, prediction interval, leave-one-out influence, and Egger-type regression. DL, DerSimonian-Laird; HKSJ, Hartung-Knapp-Sidik-Jonkman; PM, Paule-Mandel. Source: Created by the authors in Python 3.13.5 using Matplotlib 3.10.8; no external image source.

Software environment and Google Colab setup

The demonstration was executed on September 1, 2026, using Python 3.13.5, NumPy 2.3.5, pandas 2.2.3, SciPy 1.17.0, and Matplotlib 3.10.8. NumPy provides numerical arrays and operations; pandas manages study-level tabular data; SciPy provides statistical distributions and numerical optimization; and Matplotlib generates the forest plot. Google Colab provides the notebook execution environment.

# Google Colab setup !pip -q install numpy==2.3.5 pandas==2.2.3 scipy==1.17.0 matplotlib==3.10.8 import sys, numpy as np, pandas as pd, scipy, matplotlib print(sys.version) print("NumPy:", np.__version__) print("pandas:", pd.__version__) print("SciPy:", scipy.__version__) print("Matplotlib:", matplotlib.__version__)

The software environment and package versions are summarized in Table 1. The installation command recreates the reported package versions for the demonstration with any rerun; users should verify the resulting environment before rerunning the analysis.

**Table 1:** Software environment and package versions used in the demonstration

Software	Version	Role
Python	3.13.5	Execution environment
NumPy	2.3.5	Numerical arrays and calculations
pandas	2.2.3	Study-level tabular data
SciPy	1.17.0	Statistical functions and numerical optimization
Matplotlib	3.10.8	Forest plot generation
Google Colab	Cloud notebook platform	Reproducible notebook execution

Step one: data input

In current implementation, each row represents one independent two-arm comparison and does not automatically account for dependence between multiple effect estimates from the same study. For the raw-data pathway, the required fields are study identifier, intervention sample size, control sample size, intervention mean, intervention standard deviation (SD), control mean, and control SD. The direction of the effect must be defined before analysis; in this demonstration, negative Hedges g values favor the intervention. Studies that report a standardized effect estimate directly may also be incorporated when the corresponding sampling variance or standard error is available, but estimates must first be harmonized to the same metric. The required input fields and validation requirements are summarized in Table 2.

**Table 2:** Required study-level input fields and validation requirements n_t, intervention sample size; n_c, control sample size; mean_t, intervention outcome mean; mean_c, control outcome mean; sd_t, intervention standard deviation; sd_c, control standard deviation; SD, standard deviation.

Field	Meaning	Validation
study	Unique study identifier	Required; duplicates rejected
n_t	Intervention sample size	Positive integer
n_c	Control sample size	Positive integer
mean_t	Intervention outcome mean	Required numeric value
mean_c	Control outcome mean	Required numeric value
sd_t	Intervention SD	Required; >0
sd_c	Control SD	Required; >0

Step two: data validation

The script checks for missing required columns, an empty dataset, duplicate study identifiers, missing values, nonpositive or noninteger sample sizes, nonpositive SDs, and fewer than two studies. These checks are deliberately conservative: failure stops the analysis rather than silently producing pooled results. Additional clinical and extraction checks remain necessary, including duplicate cohorts, reused participants, inconsistent outcome units, incorrect treatment direction, and incorrect identification of SDs versus standard errors.

Step three: effect-size calculation

For two independent groups, the pooled within-study SD is calculated first. Cohen d is then obtained by dividing the difference between intervention and control means by the pooled SD. Hedges g is obtained by multiplying d by the small-sample correction factor J [5]. The degrees of freedom are defined below.

$J = 1 - \frac{3}{4df - 1}$

$df = n_t+n_c-2$

An approximate sampling-variance expression for Hedges g is used in this demonstration [5]. This variance expression is specific only for the assumed independent two-group demonstration. It should be replaced by a design-appropriate variance calculation for other designs and should not be interpreted as an exact or universally applicable variance formula.

$v = \frac{n_t+n_c}{n_t n_c}+\frac{g^2}{2df}$

def add_hedges_g(row): nt, nc = int(row.n_t), int(row.n_c) df = nt + nc - 2 sp = math.sqrt(((nt-1)*row.sd_t**2 + (nc-1)*row.sd_c**2) / df) d = (row.mean_t - row.mean_c) / sp J = 1 - 3 / (4*df - 1) g = J * d var_g = (nt + nc)/(nt*nc) + g**2/(2*df) return pd.Series({"yi": g, "vi": var_g, "se": math.sqrt(var_g)})

Step four: primary random-effects synthesis

The primary demonstration uses a random-effects model. Study weights are inverse-variance weights based on the sum of the within-study sampling variance and the estimated between-study variance. The DerSimonian-Laird (DL) estimator is used for the primary tau-squared estimate [6]. Because the demonstration contains 12 studies, the DL estimate is presented as a prespecified primary estimator for this demonstration rather than as a universally preferred estimator. A fixed-effect inverse-variance model is also calculated as a model-stress comparison.

Paule-Mandel (PM) is used as an alternative heterogeneity estimator. In the script, PM is obtained numerically by solving the equation shown below. The use of an alternative estimator is a sensitivity analysis rather than evidence that one estimator is universally correct [7].

$Q(\tau^2)=k-1$

Step five: confidence intervals and prediction interval

The conventional random-effects confidence interval uses a normal critical value. The sensitivity analysis uses a conservative HKSJ-style variance adjustment as defined below, with a t critical value and k - 1 degrees of freedom [8,9]. The prediction interval used the conventional approximate random-effects formulation with a t critical value based on k − 2 degrees of freedom, whereas the HKSJ-style confidence interval used k − 1 degrees of freedom. This is described as Hartung-Knapp-Sidik-Jonkman (HKSJ)-style because the prespecified variance adjustment is specific to this workflow and may differ from implementations in dedicated meta-analysis software [8,9].

$SE_{HKSJ}=\sqrt{\frac{\max\left(1,\frac{Q}{k-1}\right)}{\sum_{i=1}^{k}w_i}}$

if hksj: hk_factor = max(1.0, q / df) se = math.sqrt(hk_factor / np.sum(w)) crit = stats.t.ppf(0.975, df) else: hk_factor = 1.0 se = math.sqrt(1 / np.sum(w)) crit = stats.norm.ppf(0.975)

The prediction interval is calculated independently of the pooled-effect confidence interval. In this implementation, the prediction interval is calculated using the expression below. This separation prevents the HKSJ-style confidence-interval standard error from being inadvertently reused for prediction uncertainty. Prediction intervals address the plausible distribution of a true effect in another setting rather than the uncertainty around the average pooled effect [2,10].

$PI=\mu\pm t_{0.975,\max(1,k-2)}\sqrt{\tau^2+\frac{1}{\sum_{i=1}^{k}w_i}}$

Step six: leave-one-out influence analysis

Each study is removed in turn, the primary random-effects model is refitted, and the change from the full-data pooled estimate is calculated. The script additionally computes the absolute change and automatically identifies the study with the largest absolute change. This makes the influence conclusion reproducible rather than dependent on visual inspection. Influence is a diagnostic property; an influential study should not be removed solely because it changes the pooled estimate [11]. 

loo["absolute_change"] = loo["change_from_full"].abs() most_influential = loo.loc[ loo["absolute_change"].idxmax() ]

Step seven: small-study effects assessment

When at least 10 studies are available, the script performs an Egger-type regression of the standardized effect estimate on precision. The regression model is expressed below, with the intercept as the test statistic of interest. For standardized mean differences, the relationship between the effect estimate and its estimated standard error can complicate interpretation of conventional Egger-type regression. A statistically significant intercept is therefore treated only as a signal that may be compatible with small-study effects or funnel-plot asymmetry; it is not interpreted as proof of publication bias because asymmetry can have multiple explanations [12].

$\frac{y_i}{SE_i}=\beta_0+\beta_1\frac{1}{SE_i}+\varepsilon_i$

precision = 1 / dataframe["se"].to_numpy() standardized = dataframe["yi"].to_numpy() / dataframe["se"].to_numpy() regression = stats.linregress(precision, standardized) # The script then calculates the intercept SE and # two-sided intercept p-value explicitly.

The threshold of 10 studies is a conservative operational rule used in this framework; users should interpret small-study-effect testing in the context of the number of studies, outcome type, heterogeneity, and the methodological literature relevant to the analysis [12].

Step eight: automated robustness summary

The revised script converts the stress tests into a structured robustness summary covering direction consistency, precision assessment, influence assessment, the most influential study, heterogeneity-estimator sensitivity, and small-study effects. For the demonstration, an absolute leave-one-out change of 0.10 Hedges g or less and an absolute DL-versus-PM pooled-effect difference of 0.10 Hedges g or less are labeled stable. These are explicitly reporting conventions for the synthetic demonstration, not universal statistical thresholds.

Important assumptions and implementation limits

The framework assumes independent intervention and control groups and correctly extracted means, SDs, and sample sizes. The Hedges g sampling variance is approximate for the illustrative independent-group design. The HKSJ component is an HKSJ-style adjustment and may not reproduce every detail of dedicated meta-analysis software. The PM estimator is solved numerically. The workflow does not automatically handle dependent effect sizes, repeated samples, multilevel structures, cluster-randomized trials, missing outcome correlations, change-score correlations, or robust variance estimation. Design-specific variance reconstruction and independent statistical verification are therefore required for real clinical analyses.

Illustrative synthetic dataset

The demonstration contains 12 independent synthetic studies. The values were constructed solely to exercise the computational workflow and do not represent a real intervention, disease, population, or clinical finding. Negative Hedges g values are defined as favoring the intervention. Figure 2 displays the study estimates and the primary random-effects pooled estimate.

**Figure 2:**
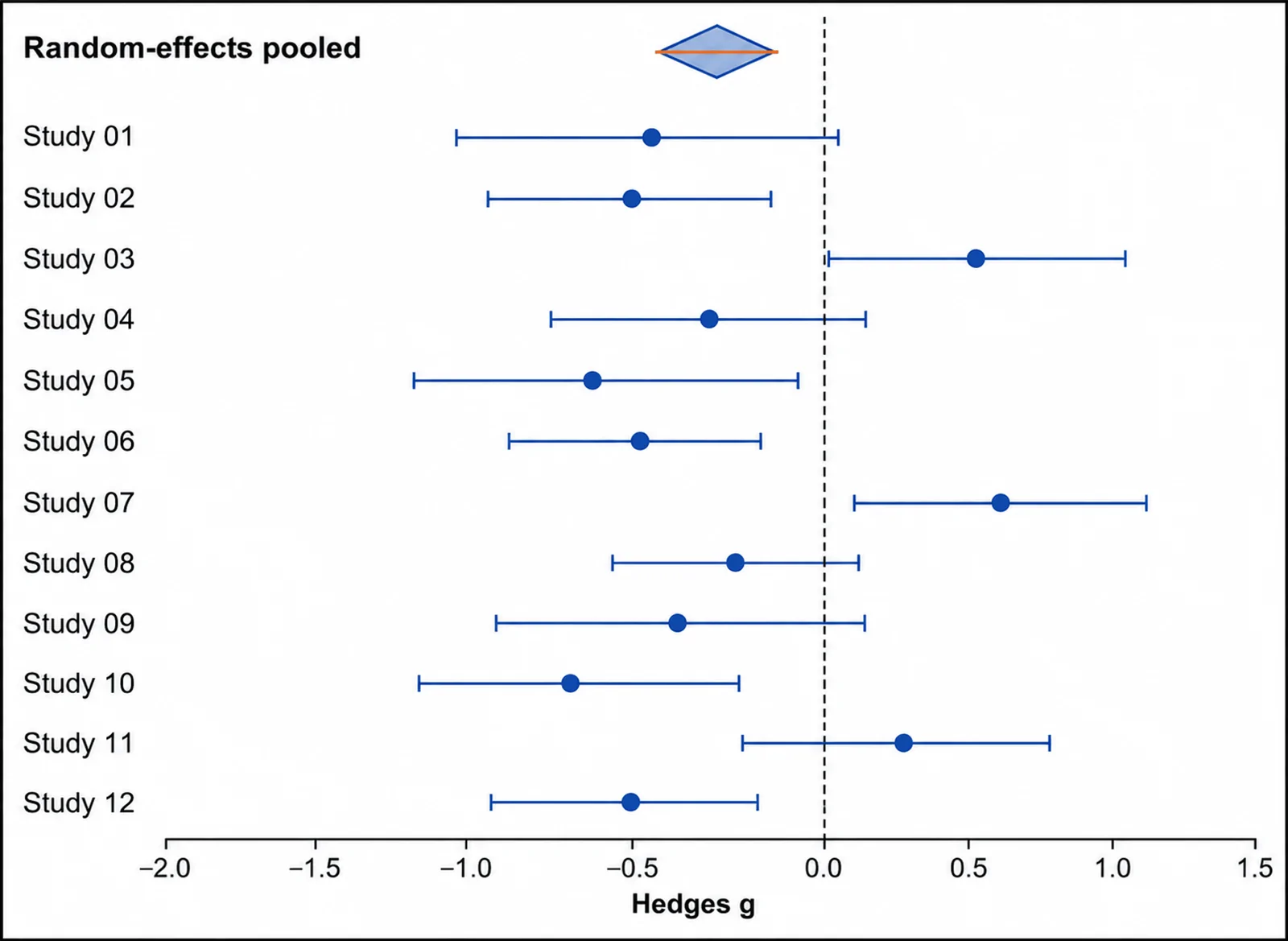
Forest plot of the illustrative 12-study continuous-outcome dataset Points represent Hedges g with 95% study-level confidence intervals; the diamond represents the primary random-effects pooled estimate. Negative values favor the intervention. Source: Created by the authors from synthetic data using Python 3.13.5 and Matplotlib 3.10.8; no external image source. CI, confidence interval.

Stress-test results

The primary and sensitivity analyses are summarized in Table 3. The primary DL random-effects model produced a pooled Hedges g of -0.249 (95% CI, -0.455 to -0.043), with tau-squared = 0.091. The 95% prediction interval was -0.961 to 0.463, indicating that the average pooled direction was negative while the plausible range for a true effect in another setting included the null value [2,10].

**Table 3:** Primary and sensitivity analyses in the synthetic demonstration CI, confidence interval; DL, DerSimonian-Laird; HKSJ, Hartung-Knapp-Sidik-Jonkman; PI, prediction interval; PM, Paule-Mandel.

Analysis	Pooled Hedges g	95% CI	Tau-squared	95% PI
Primary random-effects, DL, Wald CI	-0.249	-0.455 to -0.043	0.091	-0.961 to 0.463
Random-effects, DL, HKSJ-style CI	-0.249	-0.494 to -0.003	0.091	-0.961 to 0.463
Random-effects, PM, Wald CI	-0.248	-0.467 to -0.028	0.108	-1.023 to 0.527
Fixed-effect, inverse variance	-0.274	-0.385 to -0.163	0.000	Not calculated

The HKSJ-style CI was wider than the conventional Wald CI but retained the negative direction (95% CI, -0.494 to -0.003). The PM model produced a pooled Hedges g of -0.248 (95% CI, -0.467 to -0.028), indicating little change in the pooled point estimate despite a higher tau-squared estimate. These comparisons demonstrate model sensitivity rather than establish the superiority of one estimator [7-9].

All 12 leave-one-out analyses retained a negative pooled direction. The largest absolute change from the full-data estimate was 0.074 Hedges g after omission of Study 07. The script therefore automatically identifies Study 07 as the most influential study by the prespecified absolute-change criterion. This does not indicate that the study is erroneous or should be excluded [11]. The complete leave-one-out sensitivity analysis is provided in Appendix 1.

Because 12 studies were available, the Egger-type regression was performed. The intercept p-value was 0.352. In this synthetic example, this was not statistically significant and was not interpreted as evidence for or against publication bias. The result is appropriately treated as a sensitivity signal because asymmetry can arise for reasons other than publication bias and such tests have limited power in smaller meta-analyses [12].

Robustness reporting

The robustness summary integrates the prespecified stress tests into a structured report covering data integrity, direction stability, precision, influence, heterogeneity-estimator sensitivity, and small-study effects. The automated robustness summary is presented in Table 4.

**Table 4:** Automated robustness summary for the synthetic demonstration Stability thresholds of 0.10 Hedges g for influence and for the DL-versus-PM pooled-effect difference are reporting conventions used only for this demonstration. CI, confidence interval; DL, DerSimonian-Laird; HKSJ, Hartung-Knapp-Sidik-Jonkman; PM, Paule-Mandel.

Domain	Primary question	Demonstration result
Data integrity	Were required fields valid and uniquely identified?	Passed; analysis proceeded.
Direction consistency	Did leave-one-out estimates retain the primary direction?	12/12 leave-one-out estimates remained negative.
Precision assessment	Did HKSJ-style inference materially widen the CI?	Yes; CI widened while retaining negative direction.
Influence assessment	Did removal of one study reverse the direction?	Maximum absolute change = 0.074 Hedges g; direction remained negative
Most influential study	Which study had the largest absolute change?	Study 07; absolute change = 0.074.
Heterogeneity-estimator sensitivity	How much did DL versus PM change the pooled estimate?	Absolute difference in pooled Hedges g = 0.001
Small-study effects	Was the Egger-type intercept statistically significant?	No; p = 0.352. Interpreted only as an exploratory sensitivity signal

Implementation and reproducibility

The accompanying Python script contains the complete executable workflow. It validates the synthetic dataset; calculates Hedges g and approximate sampling variance; fits fixed-effect and random-effects models; estimates tau-squared using DL and PM; calculates conventional and HKSJ-style confidence intervals; calculates the prediction interval independently of the confidence-interval standard error; performs leave-one-out analysis; identifies the most influential study; performs the Egger-type intercept test when at least 10 studies are available; generates an automated robustness summary; exports machine-readable CSV files; and generates the dynamic forest plot. The complete script is supplied as a companion file.

The workflow is deliberately formula-transparent and therefore complements, rather than replaces, established meta-analysis software. The R packages meta and metafor provide a broader validated ecosystem of fixed- and random-effects models, meta-regression, diagnostics, and graphical functions [13,14]. For complex or high-stakes analyses, the results produced by this framework should be independently cross-checked against established software whenever feasible.

Code and materials availability

The complete executable Python code and synthetic dataset used in this technical report are freely available in a public GitHub repository [15].

## Discussion

The principal value of this workflow is that it treats robustness as a multidimensional property rather than a single sensitivity analysis. Model stress asks whether the conclusion changes when a common-effect assumption is replaced by a random-effects assumption. Heterogeneity-estimator stress examines whether the pooled estimate or uncertainty changes under an alternative tau-squared estimator. Small-sample inference stress evaluates whether an HKSJ-style adjustment materially changes the confidence interval. Influence stress determines whether the result depends strongly on one study. Small-study-effects stress evaluates whether the pattern of effect estimates is compatible with asymmetry. Considering these dimensions separately reduces the risk of presenting one favorable sensitivity analysis as proof of overall robustness [6,8,9,11,12].

Compared with established meta-analysis software, a custom Python implementation has an important transparency advantage: the formulas, validation rules, stress tests, and output files are visible in one executable workflow. The user can inspect the exact calculation used for each component rather than relying entirely on software defaults. The workflow also produces machine-readable intermediate results that can be incorporated into reproducible reporting pipelines. These features are particularly useful for teaching, auditing, and demonstrating how analytical assumptions propagate into pooled results.

The trade-off is that established software provides substantially broader functionality and more mature implementations for complex designs. The metafor and meta packages, for example, include multiple meta-analytic models, meta-regression, plotting functions, and diagnostic tools [13,14]. The present script does not automatically handle dependent effects, multilevel models, cluster-randomized trials, robust variance estimation, missing correlations, or all forms of pretest-posttest and change-score designs. A custom script can also contain programming or formula errors that may not be apparent from a single successful execution. Accordingly, this framework should be regarded as a transparent stress-testing layer rather than a replacement for established meta-analysis software.

Potential sources of error begin before the statistical calculation. Reversing the treatment direction, entering standard errors as SDs, mixing measurement units, duplicating participants from the same cohort, incorrectly reconstructing change-score variance, or applying an independent-group formula to a paired or clustered design can materially change the result. Statistical errors can also occur when analysts select an estimator after seeing results, interpret I² as a universal measure of inconsistency, or interpret a significant Egger-type test as proof of publication bias [3,4,12]. The workflow therefore cannot substitute for careful data extraction and design-specific methodological judgment.

Several recommendations follow. Users should define the clinical question and direction of benefit before data entry; maintain a data dictionary; verify each extracted value against the original report; prespecify the primary model and stress tests; record the software versions; retain all intermediate outputs; and cross-check important analyses against an established meta-analysis package. Influential studies should be investigated for methodological or clinical reasons rather than removed solely because they alter the pooled estimate [11].

The use of Hedges g also requires attention to construct comparability. Standardized mean differences are most useful when different instruments measure sufficiently similar constructs. If all studies use the same clinically interpretable scale, a mean difference may be preferable and should be selected before looking at results. Repeated-measures and change-score designs require appropriate variance calculations and should not be forced into the independent-group formula used for this demonstration [5,16].

Prediction intervals add information that a conventional pooled CI does not provide. A pooled CI may exclude zero while the prediction interval includes zero because the two intervals address different inferential targets: uncertainty around the average effect versus plausible variation in a true effect in another setting [2,10]. In the present demonstration, that distinction is visible because the primary pooled CI is entirely negative whereas the prediction interval crosses zero.

The main limitation is therefore scope. The demonstration uses synthetic data, an approximate Hedges g variance formula, a deliberately narrow set of meta-analytic models, and an HKSJ-style rather than dedicated software-specific implementation. The framework also uses reporting thresholds for influence and estimator sensitivity that are not universal statistical cutoffs. Robustness remains context-dependent. A clinically important meta-analysis additionally requires appropriate study eligibility criteria, risk-of-bias assessment, outcome harmonization, protocol transparency, and clinically justified subgroup or moderator analyses. The purpose of this report is to show how the computational portion of robustness assessment can be made explicit, reproducible, and auditable.

## Conclusions

Continuous-outcome meta-analysis is model-dependent, and robustness should be demonstrated rather than assumed. This reproducible Python framework makes data validation, effect-size calculation, heterogeneity estimation, confidence-interval stress testing, prediction, leave-one-out influence assessment, small-study-effects assessment, and automated robustness reporting explicit and is limited to independent two-arm comparisons of continuous outcomes. In the synthetic demonstration, alternative models retained the direction of the pooled estimate, Study 07 produced the largest leave-one-out change, and the prediction interval was wider than the pooled confidence interval and crossed the null value. The framework is intended to complement established meta-analysis software and to help investigators identify statistical fragility before drawing substantive conclusions.

Because the demonstration uses synthetic data, its numerical results do not support any clinical efficacy or safety conclusion.

## Appendices

**Table 5:** Leave-One-Out Sensitivity Analysis of the Primary Random-Effects Meta-Analysis The primary random-effects pooled estimate was Hedges g = −0.249. Leave-one-out analyses sequentially removed one study at a time and refit the DerSimonian-Laird random-effects model. The largest absolute change from the primary estimate occurred when Study 07 was omitted (absolute change = 0.074 Hedges g), identifying Study 07 as the most influential study under this diagnostic. All leave-one-out estimates retained the negative direction of the primary pooled effect. Influence was treated as a diagnostic of robustness and was not used as a justification for removing any study. Hedges’ g, standardized mean difference corrected for small-sample bias.

Study omitted	Leave-one-out pooled Hedges g	Absolute change from primary estimate	Interpretation
Study 01	−0.236	0.013	Primary direction retained
Study 02	−0.229	0.02	Primary direction retained
Study 03	−0.301	0.053	Primary direction retained
Study 04	−0.248	0.001	Primary direction retained
Study 05	−0.225	0.024	Primary direction retained
Study 06	−0.222	0.027	Primary direction retained
Study 07	−0.323	0.074	Largest absolute change; primary direction retained
Study 08	−0.247	0.002	Primary direction retained
Study 09	−0.237	0.012	Primary direction retained
Study 10	−0.210	0.039	Primary direction retained
Study 11	−0.286	0.037	Primary direction retained
Study 12	−0.222	0.027	Primary direction retained
